# Embracing complexity in sepsis

**DOI:** 10.1186/s13054-023-04374-0

**Published:** 2023-03-11

**Authors:** Alex R. Schuurman, Peter M. A. Sloot, W. Joost Wiersinga, Tom van der Poll

**Affiliations:** 1 Centre for Experimental and Molecular Medicine (CEMM), Amsterdam University Medical Centres - Location AMC, University of Amsterdam, Meibergdreef 9, 1105 AZ Amsterdam, The Netherlands; 2 Institute for Advanced Study, University of Amsterdam, Amsterdam, The Netherlands; 3 Division of Infectious Diseases, Amsterdam University Medical Centres, University of Amsterdam, Amsterdam, The Netherlands

**Keywords:** Sepsis, Complexity, Host response, Non-linearity, Computational models

## Abstract

Sepsis involves the dynamic interplay between a pathogen, the host response, the failure of organ systems, medical interventions and a myriad of other factors. This together results in a complex, dynamic and dysregulated state that has remained ungovernable thus far. While it is generally accepted that sepsis is very complex indeed, the concepts, approaches and methods that are necessary to understand this complexity remain underappreciated. In this perspective we view sepsis through the lens of complexity theory. We describe the concepts that support viewing sepsis as a state of a highly complex, non-linear and spatio-dynamic system. We argue that methods from the field of complex systems are pivotal for a fuller understanding of sepsis, and we highlight the progress that has been made over the last decades in this respect. Still, despite these considerable advancements, methods like computational modelling and network-based analyses continue to fly under the general scientific radar. We discuss what barriers contribute to this disconnect, and what we can do to embrace complexity with regards to measurements, research approaches and clinical applications. Specifically, we advocate a focus on longitudinal, more continuous biological data collection in sepsis. Understanding the complexity of sepsis will require a huge multidisciplinary effort, in which computational approaches derived from complex systems science must be supported by, and integrated with, biological data. Such integration could finetune computational models, guide validation experiments, and identify key pathways that could be targeted to modulate the system to the benefit of the host. We offer an example for immunological predictive modelling, which may inform agile trials that could be adjusted throughout the trajectory of disease. Overall, we argue that we should expand our current mental frameworks of sepsis, and embrace nonlinear, system-based thinking in order to move the field forward.

## Background

Sepsis is defined as life-threatening organ dysfunction caused by a dysregulated host response to infection [1]. Biomedical research has sought to dissect the pathophysiology of sepsis for decades, which has led to a rich understanding of the separate components that together manifest sepsis [2]. However, the totality of sepsis has remained ungovernable, as effective, clinically implemented therapies remain elusive. This is not for a lack of effort: numerous trials have been performed over the last 30 years, but without success. The cause of this failure is likely multi-facetted, including a disregard for heterogeneity and uncertainty, and a translational gap between pre-clinical and clinical models [3, 4]. While these considerations are relevant, we here argue for a more fundamental problem: the complexity of sepsis cannot be fully understood by single-timepoint, reductionist studies. The interplay between all components of sepsis together results in emergent behaviour that is more than and different from the sum of its parts. Emergent properties are central to complex systems, although the term ‘emergence’ can remain somewhat vague due to a variety of definitions and interpretations [5]. In essence, complex systems can display properties or behaviour that cannot be reduced to, or understood and predicted by, the individual components that make up the system. While it may feel self-evident that sepsis represents such an emergent state, still the vast majority of studies is based on a single biological snapshot of patients, often limited to a specific part of pathophysiology. In order to fully understand sepsis, it is pivotal that we complement these research efforts with approaches derived from complex systems science.

In this perspective we will discuss sepsis through the lens of complexity theory. We describe what non-linear, dynamic mechanisms contribute to the emergent state of sepsis, and how one can evaluate complex systems like the host response to infection. We highlight the progress that has been made in the understanding and analysis of complex adaptive systems and discuss why these advancements have remained generally unrecognized and have yet to translate into clinical tools. We consider the implications of treating sepsis as a complex and nonlinear system, with regards to measurement, research approaches and trial design. Specifically, we advocate more emphasis on longitudinal biological data, computational modelling and model-informed interventions.

## The complex nature of sepsis

The host response during sepsis involves the interplay between immune cells, cytokines, the coagulation cascade, the endothelial response, the complement system, the gut microbiome, the neuro-endocrine system, altered energy metabolism, the failure of whole organ systems, mechanical and pharmacological interventions by doctors, the erosive sequelae of comorbidities, one or more causative pathogens, and other factors [2]. Zooming in, the physiological components each consist of integrated molecular layers—such as the (epi)genome, transcriptome, and metabolome—that further add to the complexity of sepsis [6]. These elements are highly interconnected, often linked by nonlinear relationships; input is not necessarily proportionally related to output. Moreover, these connections are not static over time, and may depend on their spatial context within tissues and the body. Interactions can change as certain thresholds are met, feedback loops are activated or, zooming out, as disease further progresses along its natural course. Finally, this host response system is, by definition, dysregulated in sepsis. Together this delineates a spatio-temporal, complex and nonlinear system of which the resulting behaviour cannot be captured by separate analysis of the elements that it comprises. Sepsis is a typical ‘wicked’ systemic problem; by reducing it to its constituting components we lose what we are actually looking for. Indeed, while “-omics” technologies revolutionized the resolution of our snapshots of the host response during sepsis, the overall trajectory and outcome of this system has remained largely beyond our grasp.

## Complexity theory

The host response during sepsis fits the description of systems that can be analysed by complexity theory. Complex systems science seeks to understand systems that show behaviour that cannot be explained by its individual parts. Rather, the system state is shaped by intra-network dynamics, such as feedback loops or the connectivity of the system components, and external pressure on the system [7]. Perhaps counterintuitively, highly complex and dynamic systems—such as the earth’s climate, stock markets, and the human body—exist in a continuous state of non-equilibrium: they are thermodynamically open systems that display a remarkable resilience to perturbation. However, once the balance is tipped beyond a certain threshold, perturbations can rapidly propagate into profound, system-wide dysregulation. Due to path dependency, there is often no easy way to bring the system back to its original state.

Medical professionals are used to working with complexity to some extent, both on a healthcare system level [8], as well as on a biological level. While they may not label it as such, clinicians have to deal with the emergent properties of sepsis regularly: classic symptoms like vasogenic shock—inadequate tissue perfusion due to the loss of vascular tonus, temperature changes, altered states of consciousness, and coagulation disorders are examples of this. Scientifically, there have been considerable advances in the analysis and understanding of complex adaptive systems over the last decades. In the next paragraphs we will show how quickly complex behaviour and uncertainty can emerge in biological models, and the merit of methods like agent-based modelling, network analyses and cellular automata in analysing the host response. For further reference, Handel and colleagues recently published an excellent overview of different types of computational models that have been applied in the context of immunology [9].

## The emergence of complexity and uncertainty

A recent study showed how changing the amplitude of an oscillatory tumor necrosis factor (TNF, a prototypic proinflammatory cytokine) signal could generate chaotic dynamics of nuclear factor (NF)-kB expression, which resulted in a more economic production of protein complexes, and a survival benefit for cell populations [10]. The highly complex dynamics of cytokines provide another example [11]. Most cytokine interactions are nonlinear, which can lead to unpredictable results: agent-based modelling demonstrated that blood leukocytes display a nonlinear response to endotoxin, and produce chaotic NF-kB and TNF levels above a certain stimulation threshold [12]. Similarly, a computational study showed that activation of the complement system follows a nonlinear model, indicating that small initial changes can rapidly escalate into cascading and diverging trajectories [13]. These relatively simple models all reflect uncertainty—the relative degree of our inability to predict the future. Uncertainty is a fundamental feature of complex systems and, although often ignored or undervalued, highly relevant in biomedical research and healthcare [14]. The aforementioned models reflect how uncertainty already plays a role in controlled biological systems with only a few variables, which may evince how the host response to infection is inherently unpredictable: it is precisely such feedback mechanisms, nonlinear dynamics and cascading pathways that drive escalating perturbations—and uncertainty—in a patient with an infection. Of course, a treating physician also deals with variance introduced by individual genetic traits, comorbidities, medications, timing of clinical presentation, and myriad other factors.

## Computational advances in modelling and understanding immunology and sepsis

Needless to say, sepsis pathophysiology extends beyond the isolated and static models mentioned above, and involves changes over time and between tissues. Several methods can be used to capture and analyse such spatio-temporal dynamics [15]. Pigozzo and colleagues specifically modelled the response of the innate immune system to lipopolysaccharide (LPS, a bacterial product) in a dynamic computational model based on partial differential equations [16]. By including several cell types, pro-and anti-inflammatory cytokines, and the diffusion between the vascular system and tissues, the model is able to reproduce features like the temporal influx of specific cell types and the cytokine-mediated resolving of inflammation. More recent work expanded on this concept, with the incorporation of clinical, patient-derived data in order to validate computational results [17]. A different approach to incorporate spatial and temporal dynamics is the use of cellular automata, which are abstract collections of cells that have distinct states and are organized within a grid of finite number of dimensions. The states of these cells can be updated over time, in a discrete or probabilistic manner (called stochastic cellular automata), and thereby recreate interactions and organizing behaviour that can model the immune system [18, 19]. Such approaches have been used to model the microenvironment of the lung during tuberculosis infection [20], study the balance between necrosis and apoptosis in neutrophils during inflammation [21], or analyse the concerted behaviour of cell populations [22]. It is also possible to model the interaction between different sub-systems. In a two-layer model of inflammation—in which the innate immune system and parenchymal cells each oscillate, and indirectly interact through cytokines—it was shown how the complex dynamics between layers could lead to a healthy synchronized state, or a pathological state of the parenchyma [23]. Such analyses provide information on how the dynamics between sub-systems can determine state-transitions, which in a clinical sense could for instance translate as organ failure in a patient with sepsis. Network-based analyses, in which features are often represented as nodes, and connections as edges, also play a key role in understanding and visualizing complex systems. Networks can be constructed at multiple levels: for dynamic molecular intracellular pathways [24], for crosstalk [25] and interactions [26] between cells or populations of cells, or to represent the spatial organization within tissues [27]. Mostly, the combination of -omics technologies and network-based analyses has enriched our understanding of the molecular basis of immune activation on an intra- and intercellular level [28, 29]. Herein the surge in availability of high-dimensional datasets may allow for the use of machine learning techniques [30], although the “black box” nature of many of those methods makes it challenging to actually derive at functional and mechanistic insights. A combination of network-analyses with computational models, thereby mathematically defining the edges between the nodes and quantifying these interactions, could form the basis for predicting how the host might respond to infection. For instance, a recent “whole-body” computational model reproduced how systemic inflammation can cause relative hypovolemia through endothelial hyperpermeability, and the effects of fluid administration and norepinephrine hereon [31]. The model also showed differential patient outcomes when certain interventions were introduced, illustrating the potential translational and predictive value of such models. Together, these methodologies and studies each reflect considerable progress in analysing complex adaptive systems. Ideally these methods should complement and enhance each other, rather than be used in isolation [32]. One example is the combination of artificial neural networks and agent-based modelling, which has been used to predict cytokine trajectories and disease progression in (virtual) patients with sepsis [33].

## Lessons to be learned from how complexity was portrayed

More than two decades ago, Seely and Christou already stated that the host response to invading pathogens should be viewed as a complex nonlinear system, and how linear thinking may be the root of the failure of immunomodulatory trials in sepsis [34]. The authors argued for a focus on the variability and connectivity of variables, rather than the values of individual variables themselves, and an overall shift towards system-based thinking. Some years later, others proposed that a “magic bullet” therapy for sepsis is unobtainable due to the complex and chaotic nature of this syndrome [35]. Indeed, the linear analyses and crude endpoints—such as mortality—that are often used in randomized clinical trials are insufficient to model the complexity of sepsis [3, 36]. Since then, the field of complexity science has made substantial progress in analysing and understanding dynamic complex systems like the host response (as described in the paragraph above). However, despite these considerable advances, complex systems science has continued to fly under the scientific radar of the general critical care community. Why? We can identify several barriers that have contributed to this disregard for complexity: vague terminology, fundamentally different approaches to analysis, and the reality that complexity science is hard to directly channel into concrete tools.

Firstly, the informal use of the term ‘complexity’ is opaque and of itself non-explanatory, as exemplified by the Cambridge Dictionary’s definition: “Complexity: the state of having many parts and being difficult to understand or find an answer to.” To most, the statement ‘sepsis is complex’ will thus mean ‘sepsis is difficult’, and not convey the non-linearity, caveats and necessary methods that are entwined with complex systems. Imprecise use and understanding of ‘complexity’ and associated terms can have concrete negative consequences. It can lead to the impression that complexity is generally considered and examined in sepsis, while the formal, rigorous methodology that is necessary to work with complexity actually remains lacking. This in turn may impede further development of relevant methods, and widen the gap between the clinical community and fields like computational modelling and systems biology. Another point lies within the perceived dichotomy between reductionism and complexity science as an approach to study sepsis. When confronted with the limitations and biases of reductionist approaches in studying sepsis, terms like ‘intrinsic uncertainty’ or ‘irreducible complexity’ can induce a certain nihilistic stance: “the totality of sepsis is too complex to ever fully understand”. In a field that mainly seeks tangible tools with clinical benefits, this has led to an overall disregard for methods typically employed in complex systems science.

Secondly, complex adaptive systems are poorly captured by the phenomenological models that are generally practiced in biomedical research. Phenomenological models—with methods such as group comparisons, correlations and regression—try to find patterns within data [9]. The goal is to approximate causation, or to classify and predict without necessarily having to understand the exact mechanisms that underpin the system. This can be advantageous: if observational data and clinical trials identify interventions that improve patient outcome, it is not critical to exactly understand why, as long as the pros outweigh the cons. However, correlations and patterns within complex adaptive systems can be misleading, or products of very counterintuitive mechanisms. The assumption that patterns found in data are a predictor for the future behaviour of a system does not hold true for complex adaptive systems and interventions in complex systems, unless the future is fundamentally the same as the past. The state of complex systems may still be predicted [37], although the accuracy can quickly diminish as the prediction window increases. Data distributions in complex systems often follow power laws, and rarely obey Gaussian statistics. Outliers or “exceptions” have more importance, and can even drive the future dynamics of the system. Thus, complex systems require fundamentally different approaches towards analysis, generally guided more by mathematics, than usually practiced in biomedical research (see the recent opinion piece of Succi and Coveney for further reading [32]).

Finally, the task of understanding the complexity of sepsis is daunting, both in terms of scope and difficulty, and the road from complexity science to clinical tools is long. The multiplicity of components and considerations, the computational challenges and the lack of tangible tools can be discouraging. Despite these challenges, we believe it is crucial to embrace complexity science to better understand and treat sepsis. Herein, initiatives like the Society for Complex Acute Illness (SCAI, scai-med.org) have shown to be valuable in bringing together clinicians, basic scientist and computational modelers. The progress that has been made over the last decades is encouraging and will only accelerate if the concepts and methods derived from complexity science make their way into the toolkit of the general scientific community. The next paragraphs provide examples of what this may look like.

## Embracing complexity in sepsis

Analysing complex dynamic systems requires a radically different approach to measurement. Most of sepsis research is based on high-resolution snapshots of (peripheral) parameters, often focused on one or two molecular layers such as plasma proteins or the whole-blood transcriptome. Studies usually include one timepoint of measurement (for instance, the moment of admission to the intensive care unit), based on which patients are phenotyped, endotyped, clustered and randomized. Although the resolution of these measurements has undergone remarkable improvement over the last decades, dynamic processes continue to be overlooked. Such cross-sectional data will not capture the nonlinear kinetics, feedback mechanisms, and spatio-dynamic relationships that might govern sepsis (patho)physiology on a molecular level. Although there are interesting advances in estimating longitudinal dynamics from cross-sectional data [38], the inclusion of (many more) timepoints in study designs is the most robust solution. This will require a re-evaluation of how time and funding is spent: longitudinal biological monitoring is expensive, and requires a trade-off with sample size. Still, we believe that this approach towards measurement is pivotal for a fuller understanding of sepsis pathophysiology, as the resulting biological data could serve to validate and tune the computational methods that were described in the former paragraph. Integrated computational models could guide validation experiments and identify key nodes or pathways that could be targeted to modulate the emergent system state to the benefit of the host. Eventually such models could be used for pre-clinical, virtual testing of interventions, and the predictive modelling of the host response to infection. It is notable that studies utilizing continuous clinical data to study sepsis have made considerable progress, for instance by recognizing sepsis earlier with an early warning score based on machine learning of continuous electronic health record data [39]. Continuous monitoring and analysis of vital sign variability provides another example [40], with promising preliminary results regarding the diagnosis [41], outcome prediction [42, 43], and early identification of deterioration in patients with sepsis [44]. While such approaches do not necessarily elucidate the pathophysiological mechanisms of sepsis, they do illustrate the power of more continuous data. Detailed longitudinal biological data in sepsis is virtually non-existent—an indirect consequence of the overall disregard for dynamic changes—which limits comparable studies focused on the pathophysiological mechanisms of sepsis.

## Immunological predictive modelling as an example

**Fig. 1:**
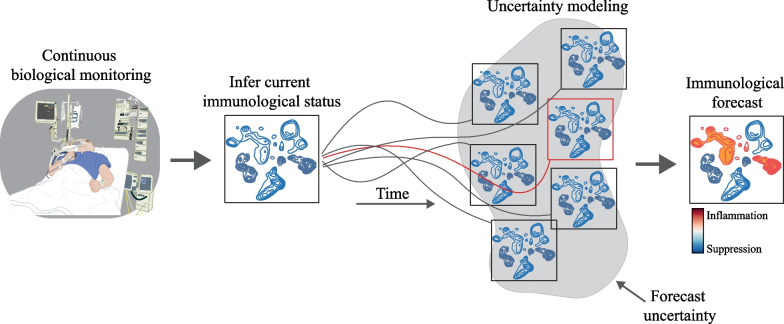
A schematic overview of immunological predictive modelling. The current immunological state of the patient is inferred from a flow of biological data. The dynamics of these data are used to generate an ensemble of predicted immunological states over time, reflecting the uncertainty of the initial state measurements and analysis errors. Forecasts are combined into a single integrated prediction, which provides a probabilistic assessment of how the immunological landscape evolves over time. The certainty of this prediction decreases as the time window increases. Concept and figure are inspired by the work of Peter Bauer and colleagues [37]

Meteorological forecasts demonstrate that, within limits, the state of dynamic complex systems can be predicted by analysis of large-scale continuous data and extrapolation from previous states [37]. Such comprehensive and integrated models of the host response are hypothetical at this point. While studies have tried to combine agent-based models with machine learning or deep reinforcement learning to model and predict sepsis [45, 46], the sparsity of continuous biological data remains a bottleneck. One could imagine ‘immunological weather stations’ that monitor a flow of multi-omics data necessary to reveal the mechanisms underlying the emergent state of the host response. Although this is technologically not yet feasible, technology has a way of outpacing expectations. Non-invasive approaches like molecular imaging are promising, in which radioisotopes, reporter genes, monoclonal antibody-based tracers and peptide-labelling can be leveraged to monitor the location, abundance, metabolism and activation status of immune cells in vivo [47]. Other read-outs, like the dynamic velocity of immune cells in the circulation through time-lapse MRI [48, 49], or the tracking of bacterial populations throughout the body [50], are also possible. Embedding such dynamic, patient-derived data into an integrative computational model of the host response could help to infer and predict the patient state during sepsis. As a concrete example of how this may look, we recently published a quantitative computational model of systemic inflammation in patients undergoing cardiothoracic surgery [17]. The model was calibrated and validated with clinical data, and was able to predict the dynamic responses to certain interventions. In Fig. 1 we provide a different example of predictive immunological modelling, based on the uncertainty modelling that is practiced in meteorology [37]. It is important to realize that immunology is only part of a larger system, and that components like coagulation, hormones and energy metabolism are still ignored. A fully comprehensive model will require a huge multidisciplinary effort to materialize, combining computational, experimental and clinical work.

## Towards trials and clinical applications

A more holistic view of sepsis pathophysiology should impact how we design trials. If we assume that the most important approach—aside from supportive care—to improve clinical outcome is modulation of the dysregulated host response, then we must first understand how we can shape the behaviour of this system to improve clinically relevant outcomes. From a complexity theory point of view, it is highly unlikely that a single, invariable intervention can do this, or let alone influence crude outcome measures such as mortality. Ideally, clinicians would only ‘nudge’ specific parts of the host response during (or prior to) sepsis, in order to bias the system towards a return to homeostasis. But when and where to push? Due to a general lack of longitudinal data it remains unclear when an adequate response to infection becomes a dysregulated one; the tipping point of sepsis is still unknown. With appropriate longitudinal data, complexity modelling may help to recognize instability in an early phase and identify when to act. Based on continuous monitoring of the variability of vital parameters, predictive instability modelling [51] and machine learning-based methods [39] have shown to identify patient deterioration in an early stage. Such methods might also be applied on biological data, if these were available. Furthermore, the goal, the state we want the host response to be in, is yet undefined. To pursue a return to normal homeostasis is to ignore the constraints of the disease, which most likely requires a dynamic host response throughout its course to be controlled. An appreciation for dynamic changes may inform more ´agile´ trials, which can be designed to be adjusted throughout the trajectory of the disease. Patient-derived biological data could be collected at multiple timepoints and combined with a computational model, based on which the intervention can be modulated. Such an approach would not require a fully comprehensive integrated computational model of the host response, which is unattainable at the moment, but rather a computational tool that specifically models the mechanisms that are targeted by the intervention.

These ideas are not new. Already in 2004 the concept of in silico clinical trials was introduced and evaluated in the context of sepsis. In one study, agent-based modelling of the innate immune response was used to simulate the effects of various anti-cytokine therapies to virtually treat a systemic inflammatory response syndrome, revealing that none of the tested therapies resulted in improved system survival [52]. In a different setup, Gilles Clermont and colleagues constructed a mechanistic model of the acute inflammatory response using differential equations, and subsequently designed a clinical trial to test several anti-TNF regimens in 1000 virtual patients in which several host and pathogen properties were varied to introduce heterogeneity [53]. With this approach the authors found that anti-TNF benefitted one subgroup of patients, but harmed another, an effect that was strongly dependent on the dose and duration of the therapy. Similarly, virtual ‘agile’ trials—using predictive modelling to seek a control strategy to continuously push a system towards an optimal state or trajectory—have suggested that successful therapies in sepsis will require a multi-target strategy that varies in dosage and timing [45, 54, 55]. Despite these and other computational advances [15, 56], it is dispiriting to note that now, almost two decades later, the role for computational modelling in clinical trial design remains minimal. To change this, we must overcome the barriers we previously described, calibrate and validate computational models with detailed longitudinal biological data collected in relevant clinical settings, and bring together clinical and computational disciplines.

## Conclusions

While clinical applications may be decades away, the main point is this: the host response during sepsis is an extremely complex and nonlinear process, which can result in emergent behaviour that cannot be captured by single-timepoints and isolated analyses of specific host response features. While this may perhaps feel obvious, the reality is that the vast majority of sepsis research is still based on such measurements. A fuller understanding of the emergent behaviour of sepsis pathophysiology warrants a revision of how biological measurements are currently collected and analysed. This will require a reappraisal of how focus and funds are currently allocated; longitudinal or continuous sampling is labour-intensive and costly. However, such data will facilitate a more prominent role for techniques like dynamic computational models and network-based analyses, which have the capacity to capture mechanisms that would otherwise be missed. In turn, this can inform more dynamic trials that seek to influence treatable traits by continually pushing the host response to a more beneficial state. In this Perspective we mostly advocated a shift towards longitudinal biological data collection, but more is needed. It is hard to overstate the importance of rigorously characterizing, defining and modelling the host response, the system that we as clinicians seek to influence, in explicit and clear terms. This will help clarify the merits and limits of our current methods and approaches, and pave the way towards the endgame: altering the disease trajectory of a patient with sepsis through targeted, model-informed interventions [15, 56].

The complexity of sepsis pathophysiology requires us to expand our current mental frameworks, and embrace nonlinear, system-based thinking in order to move the field forward.

## Data Availability

Not applicable.
